# Feasibility and outcome of genomics-guided treatment selection in advanced cancer – the MEGALiT explorative clinical trial

**DOI:** 10.2340/1651-226X.2025.43366

**Published:** 2025-06-04

**Authors:** Lars Ny, Henrik Fagman, Johan Botling, Loviisa Mantovaara, Peter Asplund, Hannah Karlsson, Jennie Aust, Edvard Abel, Mats Hellström, Joakim Crona, Peter Nygren

**Affiliations:** a Institute of Clinical Sciences, Department of Oncology, University of Gothenburg, Gothenburg, Sweden; b Department of Oncology, Sahlgrenska University Hospital, Gothenburg, Sweden; c Department of Clinical Pathology, Sahlgrenska University Hospital, Gothenburg, Sweden; d Department of Laboratory Medicine, Institute of Biomedicine, Sahlgrenska Center for Cancer Research, University of Gothenburg, Gothenburg, Sweden; e Department of Immunology, Genetics and Pathology, Uppsala University, Uppsala, Sweden; f Department of Blood and Tumor Diseases, section of Oncology, University Hospital, Uppsala, Sweden; g Department of Medical Sciences, Uppsala University, Uppsala, Sweden

**Keywords:** precision cancer medicine, genomics, clinical trial, targeted drug, PD-L1 inhibitor

## Abstract

**Background:**

Precision cancer medicine (PCM) is key to advancing cancer treatment beyond the standard of care. We performed an explorative clinical trial, MEGALiT, to investigate the feasibility, safety, and clinical benefit of genomics-based PCM in advanced cancer.

**Methods:**

MEGALiT recruited adult patients with advanced solid tumors refractory to standard treatment. Tumor DNA from newly acquired biopsies or ctDNA were analyzed for alterations targetable with the PD-L1 inhibitor atezolizumab, the MEK inhibitor cobimetinib, the mTOR inhibitor everolimus, or the PARP-inhibitor niraparib. Any other ‘in study’ treatment was left to the discretion of the physician.

**Results:**

Outcome data are reported for 153 patients. The median age was 65 years and the most common diagnoses were colorectal, prostate, and ovarian cancer. The median time from study inclusion to the Molecular Tumor Board was 35 days for tumor sampling by biopsy and 21 days by ctDNA. Of the 44 patients allocated to a study drug, 38 started treatment. The median follow-up was 1.9 years. Of the patients on a study drug and evaluable for tumor response, 6% (2/32) had partial remission, and 25% (8/32) had disease control at 16 weeks. Median overall survival for patients starting a study drug was longer, 7.4 months, compared to 2.7 months for the 61 untreated patients (HR 0.43; log-rank *p* < 0.0001), but shorter than for the 50 patients receiving treatment of physician’s choice, 11.8 months (HR 0.55; log-rank *p* = 0.012). No significant procedure- or drug-related severe adverse events were observed.

**Interpretation:**

Genomics-guided treatment selection in advanced cancer is feasible and safe. However, evidence of patient benefit warrants further investigation.

## Introduction

Rapid advances in cancer biology and analytical methods have enabled the development of an arsenal of ‘targeted’ anti-cancer drugs. Genome-driven cancer treatment involves the stratified use of targeted drugs to exploit specific tumor gene biomarkers [1], aiming to maximize efficacy and treatment benefits for patients through the concept of precision cancer medicine (PCM) [2].

Nevertheless, gene biomarker-drug activity relationships are context-dependent and vary between tumor diagnoses [1]. Thus, the utility of a genomic biomarker needs to be established based on clinical outcomes data and the extent to which such data allow for hierarchy classifications. The ESMO Scale for Classification of Actionability of molecular Targets (ESCAT) [2] is used to prioritize gene biomarkers in patient selection for targeted therapy. ESCAT tier I or II indicate clinical relevance and tiers III–V potential clinical relevance or preclinical evidence only.

The number of gene biomarkers–drug relationships established has fueled a hope that biomarker driven therapies could improve cancer treatment when anti-cancer drug selection is mainly based on the genomic biomarker status. This approach has been evaluated in several clinical trials, typically recruiting patients with advanced cancer progressing while on standard treatment [3–13]. The fraction of patients included starting genomics guided treatment varies widely (6–56%) as does tumor response rates among those starting such treatment (11–50%), depending on molecular targets and drug access [4, 6, 8–13]. Randomized trials comparing this concept with the standard of care have not shown significantly improved outcomes [14–16]. However, a recent preliminary report of a randomized trial in an earlier line of treatment indicates superiority of genomics-guided treatment [17].

With this background, the aims of MEGALiT, A MolEcularly Guided Anti-cancer drug off-Label Trial, were to acquire experience from genomics-based PCM with respect to the feasibility of the analytical part and assess evidence of the potential patient benefits from the concept.

## Methods

### Study overview

MEGALiT (NCT04185831) was a prospective open-label, non-randomized, combined basket and umbrella explorative clinical (phase 2) trial conducted at two centers, Uppsala University Hospital and Sahlgrenska University Hospital, Sweden. It investigated the efficacy and safety of genomics-guided therapy with commercially available targeted anti-cancer drugs in patients with advanced solid tumors refractory to standard therapy. The trial had four parallel drug baskets: the mitogen-activated protein kinase (MEK) inhibitor cobimetinib, the poly (ADP-ribose) polymerase (PARP) inhibitor niraparib, the mammalian target of rapamycin (mTOR) inhibitor everolimus, and the programmed death-ligand 1 (PD-L1) blocking antibody atezolizumab. Individual patient treatment allocation was based on results from the genomic analysis discussed at the study Molecular Tumor Board (MTB). The study was planned to include 154 patients, which was estimated to result in the allocation of eight patients per drug basket.

### Patients

Patients ≥ 18 years old with metastatic or advanced solid tumors who had progression on the last line of established therapy were eligible to participate if they had normal major organ function, a WHO performance status of 0–2, and expected to be clinically stable for at least 4 weeks. Patients were required to have a tumor lesion suitable for sampling by biopsy and/or archived tumor material available for molecular analysis.

### Study objectives

The primary objective was to assess the objective response rate (ORR) and tumor control rate of genomics-guided therapy using objective tumor response rate and stable disease as endpoints. The secondary objectives were: (1) clinical feasibility of genomics guided therapy selection, defined as the proportion of patients included with actionable genomic analysis results within 28 days from inclusion; (2) the anti-tumor activity of the study drug compared with the line of therapy before study start; (3) drug and procedure related safety; and (4) to compare the outcome of patients starting study drug treatment with that of patients included but not treated with a study drug.

### Outcome assessments

Tumor treatment response was assessed radiologically by contrast-enhanced computed tomography (CT) every 2nd month or, for prostate cancer, by serial measurement of prostate-specific antigen (PSA). Disease control at 16 weeks included patients with stable disease or tumor response up until this time point.

Progression-free survival (PFS) was defined as the time from the start of treatment until radiological progression according to RECiST 1.1 or iRECiST if applicable, a PSA elevation of ≥ 25%, obvious clinical disease progression, or death. Efficacy data on tumor response and PFS on the treatment just prior to study inclusion and for any treatment after the one given in association with the trial was retrieved from electronic medical records. Overall survival (OS) was defined as the time from study inclusion to death from any cause. Drug-related safety and adverse events were graded according to the National Cancer Institute (NCI) Common Terminology Criteria for Adverse Events (CTCAE) v4.03.

### Molecular tumor analysis, MTB, criteria for treatment allocation, and treatment

The trial was initially planned to exclusively use newly acquired tumor tissue obtained through ultrasound-guided biopsy for centralized genomic analysis on the Foundation Medicine platform (F1CDx; Penzberg, DE). A feasibility assessment performed midway through the study indicated frequent failure to obtain representative tumor material by biopsy and limited capacity for the biopsy procedure. Based on these considerations, it was decided to allow the use of blood sampling for circulating tumor DNA (ctDNA) analysis using the FoundationOne Liquid CDx (F1LCDx). Thus, ctDNA became the dominating sampling technique in the second half of the trial. In patients where the centralized analysis did not fulfill F1CDx quality criteria or when ctDNA analysis was considered not conclusive, a local analysis of tumor tissue on the TSO500 [18] or, subsequently, the GMS560 platform (300‐600 genes) [19] was used.

Genomics reports were discussed at the study MTB convening every 2 weeks. The MTB was attended by oncologists, pathologists, molecular pathologists and study nurses. The results were interpreted with respect to criteria for treatment allocation within the trial as follows. In general, a potentially actionable predictive biomarker for treatment allocation was defined (1) as a molecular alteration known to promote tumor growth or progression, (2) as the target of a drug, (3) a specific eligibility criterion for enrollment on a clinical trial, or (4) its known ability to confer sensitivity to a drug.

In the first half of the trial, treatment allocation was based strictly on the presence of an oncogenic germline or somatic mutation in *ATM*, *BRCA1* or *BRCA2* for the PARP inhibitor niraparib; in *NF1* or *MAP2K1* for the MEK inhibitor cobimetinib; in *MTOR*, *TSC1* or *TSC2* for the mTOR inhibitor everolimus; and ≥ 7 non-synonymous somatic mutations per megabase for the PD-L1 antibody atezolizumab.

The interim feasibility assessment indicated that fewer patients than expected were allocated to a study drug using the above criteria. Pathway core consensus components were then revised and widened for everolimus, cobimetinib, and niraparib. A consolidated list of candidate genes for each pathway created by taking the union of pathway members across multiple TCGA studies and then further curating them based on updated literature, public pathway databases, and expert opinion [20]. For atezolizumab, the criterion was unchanged, i.e. ≥ 7 non-synonymous somatic mutations per megabase. Details on the revised treatment criteria are provided in Supplementary Table 1.

Patients with tumors lacking genomic alterations targetable by any of the study drugs were cared for by the physician in charge of the patient without guidance from the study protocol or study management team and were followed for survival and, as applicable, effect of physician’s choice of treatment, labeled treatment ‘while in study’.

The conclusion from the MTB was either a recommendation to start treatment with a study drug or that there was no suitable study drug. The genomics reports were available for the physician in charge of the patient.

Patients were evaluated for treatment response every 2 months, and treatment was continued until objective disease progression, clinical deterioration, serious adverse events, or patient withdrawal of consent. Dose reductions and treatment interruptions due to adverse events followed principles detailed in the labeling for each drug.

### Statistics

Patients’ characteristics are presented with descriptive statistics. Comparisons between groups were made with Fisher’s exact test or the Kruskal–Wallis test. Treatment outcome is reported for all patients starting treatment irrespective of time on treatment. Survival data were presented according to Kaplan–Meier and analyzed using a log-rank test with hazard ratios with 95% confidence intervals. Statistical inference was two-sided throughout and *p* < 0.05 was considered statistically significant.

### Ethics

The study was conducted in accordance with the international standards of ICH-GCP (Good Clinical Practice) guidelines, including study monitoring and study drug accountability, and the Declaration of Helsinki (2013 version available at the study start). Each patient provided informed consent for all study procedures. The study was approved by the Swedish ethics review authority (Dnr 2019-04236).

## Results

From November 2020 to August 2023, 148 unique patients were included: 81 in Uppsala and 67 in Gothenburg. In accordance with the protocol, five patients were included twice based on two different tumor samplings, with treatment until progression in between. These patients were regarded as unique patients at each inclusion but were censored for OS at the time of the second inclusion. Thus, the study outcome is reported for 153 patients.

The median patient age was 65 years (range 21–84), and 86 (56%) of patients were female (Table 1). At the time of inclusion, the majority of patients had a WHO performance status of 0 or 1 and a median of four lines of prior treatment for advanced disease (range 0–14). Two patients had no prior systemic treatment: one patient with cervical carcinoma and Fanconi syndrome and one with atypical meningioma progressing after radiotherapy. The three most common cancer diagnoses among included patients were colorectal (18%), prostate (15%) and ovarian cancer (12%).

**Table 1:** Number of patient inclusions, patient characteristics and cancer diagnosis; total and divided into subgroups analyzed.

Characteristic	All inclusions (*n* = 153)	Allocated to study drug, treated* (*n* = 38)	Not allocated to study drug (*n* = 109)	Not allocated to study drug, treated while in study (*n* = 50)**	Not treated while in study (*n* = 61)***	Comparison of characteristics between groups
Age;						Not significant
Median	65	64	65	65	66	
Range (years)	(21–84)	(21–83)	(26–84)	(26–84)	(28–81)	
Sex; *n* (%)						Not significant
Females	86 (56)	25 (66)	56 (51)	29 (58)	30 (49)	
Males	67 (44)	13 (34)	53 (49)	21 (42)	31 (51)	
WHO performance status at inclusion; *n* (%)						*p* = 0.0063 allocated, treated vs not treated *p* = 0.0393 not allocated, treated vs not treated
0	57 (37)	18 (47)	38 (35)	21(42)	15 (25)	
1	49 (32)	15 (40)	29 (26)	11 (22)	23 (38)	
2	17 (11)	0 (0)	17 (16)	5 (10)	12 (20)	
3	3 (2)	0 (0)	3 (3)	1 (2)	2 (3)	
No data available	27 (18)	5 (13)	22 (20)	12 (24)	9 (15)	
Lines of treatment prior to inclusion; *n* (%)						*p* = 0.0255 allocated, treated vs not treated *p* = 0.0501 not allocated, treated vs not treated
0	2 (1)	0 (0)	2 (2)	2 (4)	0 (0)	
1	7 (5)	1 (3)	6 (5)	5(10)	1 (1)	
2	28 (18)	4 (11)	21 (21)	6 (12)	17 (28)	
3	33 (22)	7 (18)	25 (22)	5(10)	20 (33)	
≥ 4	83 (54)	26 (68)	55 (50)	32 (64)	23 (38)	
Median (range)	4 (0–14)	4 (1–14)	3 (0–13)	4 (0–13)	3 (1–7)	
**Cancer diagnosis (*n*)**						
Colorectal	27	6	19	4	17	
Prostate	23	3	20	12	7	
Ovarian	18	8	10	9	1	
Pancreatic	17	0	16	3	13	
CC	10	1	8	2	6	
Uterine cervix	9	4	5	4	1	
NET	7	2	5	2	3	
Melanoma	4	1	2	1	2	
Breast	6	2	3	3	1	
Uterine body	3	2	1	1	0	
Salivary gland	2	1	1	1	0	
Glioblastoma	4	0	4	0	4	
Low grade brain tumor	2	0	2	1	1	
CUP	3	0	3	1	2	
Endocrine cancer	3	1	2	1	1	
GI-cancer except colorectal	5	4	1	1	0	
Other	10	3	7	4	2	

* Six patients were allocated to a study drug but did not start any treatment.

** In four patients not allocated to a study drug, treatment status while in study is unknown.

*** Includes six patients allocated to a study drug but did not start any treatment, and excludes four patients not allocated to a study drug with treatment status while in study unknown.

CC: cholangiocarcinoma; NET: neuroendocrine tumor; CUP: cancer of unknown primary; GI: gastrointestinal.

Tumor sampling was performed from a newly acquired biopsy in 66 cases, from archived biopsy material, mostly obtained prior to systemic therapy, in 11 cases, and by ctDNA in 76 cases. Results for the MTB were available in 132 cases (86%). Biopsies failed in 10 cases due to the tumor cell fractions being too low or not providing representative material, and in 11 cases planned biopsies were not performed for safety reasons. Fifteen biopsies not fulfilling quality criteria in the centralized analysis were successfully analyzed locally. All analyses based on ctDNA were reported back for assessment at the MTB. However, in 17 cases (22%), the genomics results were not considered for treatment allocation due to low tumor fraction or allele frequencies; in 12 of these cases, analysis was then performed on archived tumor tissue.

The median time from study inclusion to MTB was 28 days (range 11–168). For tumor sampling by biopsy, the median time was 35 days (range 14–168), with 32% having results available to the MTB within 28 days. For sampling by ctDNA, the corresponding values were 21 days (range 11–64) and 75%, respectively.

**Figure 1:**
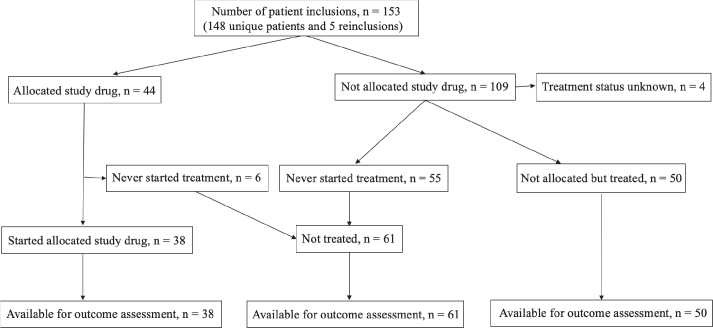
Overview of patient treatment status while in study.

Based on the MTB assessment of the gene analysis results, 44 patients (29% of those included) were allocated to a study drug within the trial and 38 (25%) started treatment (Figure 1 and Table 1). Six patients allocated to a study drug did not start treatment due to clinical deterioration. Of the 109 patients not allocated to a study drug, 50 received treatment of physician’s choice during the study, whereas 61 patients had no further treatment after study inclusion. Patients allocated to a study drug treatment or treatment of physician’s choice had better performance status and a greater number of treatment lines prior to study inclusion compared with those not treated and tumor diagnoses also differed between the groups (Table 1).

Eight patients started study drug treatment based on tumor sampling by biopsy and the original criteria for treatment allocation and 30 started based on sampling by ctDNA and the revised criteria. Ovarian, colorectal, or uterine cervical cancer were the most common diagnoses treated with a study drug. Atezolizumab was the most common drug allocated to 22 patients, of whom 18 started treatment, followed by cobimetinib (15/13), niraparib (5/5), and everolimus (2/2). Allocation and treatment start per diagnosis are detailed in Table 2. The median time from the MTB to the start of study drug treatment was 26 days (range 3–125).

**Table 2:** Number of patients allocated/starting treatment within trial in total and divided on diagnosis and basket.

Number allocated/treated and as per diagnosis and basket	Atezolizumab	Cobimetinib	Niraparib	Everolimus
Total: 44/38	22/18	15/13	5/5	2/2
CRC	6/4	3/3	0/0	0
Cervix	3/3	0	0	1/1
Prostate	1/1	2/2	0	0
Ovarian	4/4	4/4	0	0
Uterine body	2/2	0	0	0
Breast	1/0	1/1	1/1	0
Cholangiocarcinoma	1/0	0	1/1	0
Pancreatic	0/0	1/0	0	0
Esophagus	1/1	0	0	0
Gastric	1/1	0	0	0
Glioma	0	0	1/1	0
Pancreatic NET	1/1	0	0	1/1
NEC	1/1	0	0	0
Mesothelioma	0	0	1/1	0
Melanoma	0	2/1	0	0
NSCLC	0	0	1/1	0
Salivary	0	1/1	0	0
Vaginal	0	1/1	0	0

Patient diagnosis in total and as per drug basket; numbers allocated/started.

CRC: colorectal cancer; NET: neuroendocrine tumor; NEC: neuroendocrine cancer; NSCLC: non-small cell lung cancer.

Among patients not allocated to a study drug but starting treatment of physician’s choice, the majority were treated with targeted drugs or combinations thereof (Supplementary Table 2). The selection of the targeted drug was seemingly influenced by the genomics analysis in about half of the cases, as judged from the physicians’ treatment considerations in the patient records at the time of treatment start.

Molecular biomarkers were ESCAT tier 1 in 47% in the study drug group, all for atezolizumab and the predefined cut-off of ≥ 7 non-synonymous somatic mutations per megabase, and tiers III–V in 53%, all for cobimetinib, niraparib and everolimus. Applying the more conservative cut-off of ≥ 16 mutations per megabase for atezolizumab [21], tier 1 in the study drug group was reduced to 13%. Molecular biomarkers used for study drugs selected in patients starting treatment are detailed in Supplementary Table 3. Molecular biomarkers in patients treated according to physician’s choice were tier I in 8% and the remaining unclassified.

Tumor response on study drug treatment was evaluable in 32 patients, whereas six patients were not evaluable or data were missing (Table 3, detailed in Supplementary Table 4). Two patients (6%) had partial response: one with uterine cervical cancer on atezolizumab based on TMB 12 mutations/Mb, and one with ovarian cancer on cobimetinib based on a *BRAF* V600E mutation. Seven patients (22%; one on atezolizumab, four on cobimetinib, and two on niraparib) had stable disease as the best response, and 23 (72%) had progressive disease. The disease control rate at 16 weeks from treatment start was 25% (eight patients). For treatments of physician’s choice, these figures were somewhat although not statistically significantly better (*p* = 0.3442, comparing PR + SD vs. PD); 6% had PR, 33% had stable disease, and 61% had progressive disease as the best response. The disease control rate at 16 weeks was 28%.

**Table 3:** Best overall response rate and DC16w per treatment line and study subgroup.

Treatment line	Allocated to study drug, treated; *n* (%)	Not allocated to study drug, treated while in study; *n* (%)	Not treated while in study; *n* (%)	Between group comparison
Treatment while in study	CR: 0	CR: 0 (0)	Not applicable	*p* = 0.3442
	PR: 2 (6)	PR: 3 (6)		
	SD: 7 (22)	SD: 15 (33)		
	PD: 23 (72)	PD: 28 (61)		
	DC16w: 8 (25)	DC16w: 13 (28)		
	Not evaluable/no data available: 6	Not evaluable/no data available: 4		
Treatment immediately prior to study inclusion	CR: 0	CR: 0	CR: 0 (0)	*p* < 0.0001
	PR: 9 (24)	PR: 8 (17)	PR: 4 (8)	
	SD: 14 (38)	SD: 23 (52)	SD: 10 (19)	
	PD: 14 (38)	PD: 13 (30)	PD: 38 (73)	
	DC16w: 21 (57)	DC16w: 25 (57)	DC16w: 10 (19)	
	No data available: 1	No data available: 4	No data available: 9	
Treatment immediately after that while in study	CR 0 (0)	CR: 0 (0)	No data available: 1	*p* > 0.9999
	PR: 0 (0)	PR: 1 (8)		
	SD: 5 (42)	SD: 3 (25)		
	PD: 7 (58)	PD: 8 (67)		
	DC16w: 5 (42)	DC16w: 4 (33)		
	No data available: 4	No data available: 2		

CR: complete response; PD: progressive disease; PR: partial response; SD: stable disease; DC16w: disease control at 16 weeks (includes CR, PR, and SD).

Response rates for the treatment immediately prior to study inclusion varied between groups (*p* < 0.0001), with inferior results for patients not treated while in study (Table 3). A few patients had a subsequent line treatment after progression on the study drug or the treatment of physician’s choice, with no significant difference in the best response between groups (*p* > 0.9999).

**Figure 2:**
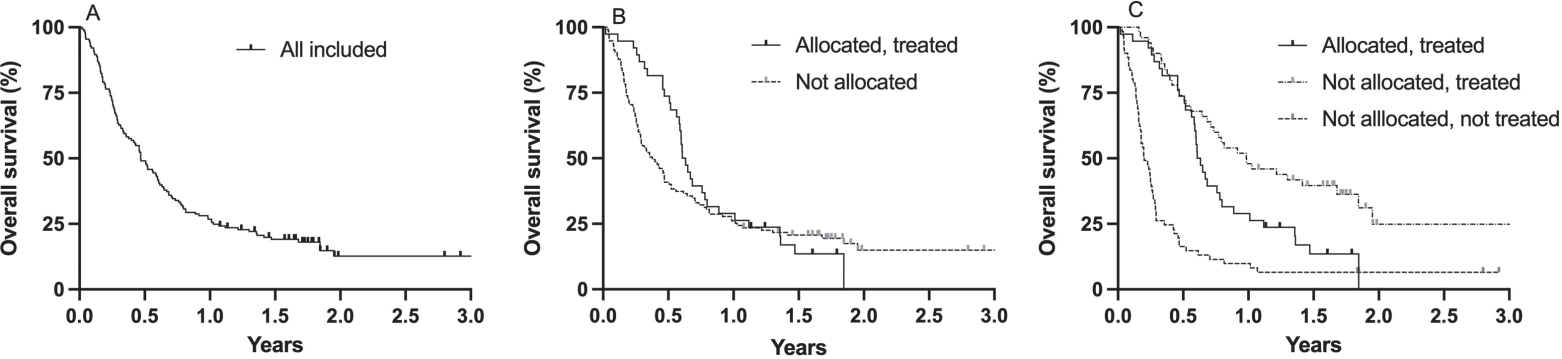
Kaplan–Meier estimates of overall survival in all patients included (A); patients allocated to a study drug and treated and patients not allocated to a study drug (B); patients allocated to a study drug and treated, patients not allocated to a study drug but treated, and patients not treated while in study (C).

Median follow-up was 1.9 years (range 1.1–3.9). Median OS for all patients included was 5.6 months (95% CI 4.3–7.3; Figure 2A). Median OS for patients allocated to a study drug and treated was longer than for patients not allocated to a study drug, but not statistically significantly so; 7.4 months (95% CI 6.8–9.6) compared with 4.5 months (95% CI 3.4–6.1; log-rank HR 0.84; 95% CI 0.58–1.23, *p* = 0.399; Figure 2B). Median OS for patients not allocated to a study drug but treated based on physician’s choice was 11.8 months (95% CI 8.2–17.4), significantly longer than for patients allocated and treated with a study drug (log-rank HR 0.55; 95% CI 0.33–0.90, *p* = 0.012) and patients not treated (median OS 2.7 months; 95% CI 2.1–3.3: log-rank HR 0.29; 95% CI 0.19–0.45, *p* < 0.0001; Figure 2C). Compared with patients not treated, OS was also significantly longer for patients treated with a study drug (log-rank HR 0.43; 95% CI 0.28–0.65, *p* < 0.0001; Figure 2C).

**Figure 3:**
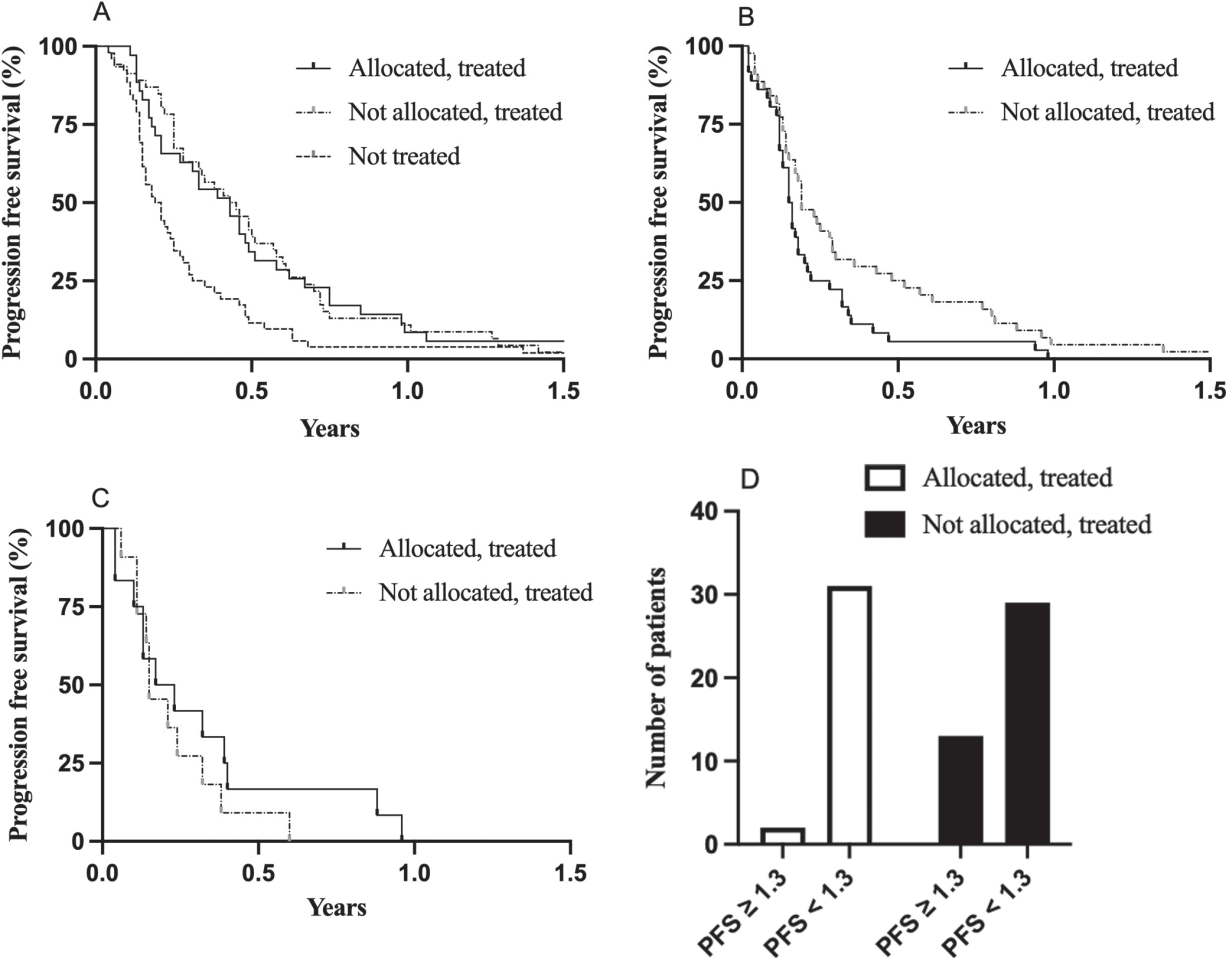
Progression-free survival (PFS) for the indicated patient groups for treatment immediately prior to study inclusion (A), treatment while in study (B), and treatment after the study (C). Panel D shows number of patients with a PFS within study/PFS prior to study inclusion ratio of ≥ and < 1.3.

Median PFS on treatment prior to study inclusion in patients allocated to a study drug was 5.2 months (95% CI 2.5–6.1) and essentially identical in patients not allocated but treated (5.3 months; 95% CI 3.4–7.0; log-rank HR 1.04; 95% CI 0.67–1.62, *p* = 0.841; Figure 3A). It was shorter in patients not treated (median 2.4 months; 95% CI 1.8–3.0) compared with patients allocated and treated (log-rank HR 1.8; 95% CI 1.2–2.7, *p* = 0.0044; Figure 3A).

While in study, PFS was longer in patients not allocated but treated based on physician’s choice compared with patients allocated and treated with a study drug; median PFS 2.3 months (95% CI 1.8–3.5) compared with 1.9 months (95% CI 1.4–2.4; log-rank HR 0.59; 95% CI 0.37–0.96, *p* = 0.0346; Figure 3B). A few patients from these groups had a subsequent line of treatment while in the study with median PFS of 2.4 months (95% CI 1.2–4.8) and 1.8 months (95% CI 1.3–4.6; log-rank HR 0.71; 95% CI 0.31–1.64, *p* = 0.3831; Figure 3C) for patients in the study drug and physician’s choice of treatment groups, respectively.

Among patients allocated and treated, two out of 33 patients (6%) with data available had a ratio of PFS within/PFS prior to the study of ≥ 1.3, compared with 13 out of 42 patients (31%) on physician’s choice of treatment (*p* = 0.0087; Figure 3D).

There were no grade ≥ 3 AEs considered at least possibly related to tumor sampling by biopsy or ctDNA (Table 4). There were 10 grade ≥ 3 AEs in 10 patients considered at least possibly related to trial drug treatment, six for atezolizumab and four for cobimetinib; none were considered serious or unexpected.

**Table 4:** Grade ≥ 3 adverse events considered possibly related or related to study procedures and specific to study drug treatment.

Type of event	Grades 3–4
*Procedure related*	
Tumor biopsy (*n* = 77)	None
ctDNA (*n* = 76)	None
*Drug related*	
Atezolizumab (*n* = 17)	Autoimmune hepatitis 1
	Myositis 1
	Elevated liver enzymes 2
	Abdominal pain 1
	Fatigue 1
Cobimetinib (*n* = 12)	Stop in stoma 1
	Nausea/vomiting 1
	Anemia 1
	Bullous pemphigoid 1
Niraparib (*n* = 5)	None
Everolimus (*n* = 1)	None

ctDNA: circulating tumor DNA.

## Discussion

Genomics-guided selection of treatment with everolimus, cobimetinib, niraparib or atezolizumab outside of their approved labeling was found feasible and safe in patients with therapy refractory advanced solid tumors. Treatment with a study drug was associated with improved OS compared with patients not treated while in study. However, the tumor response rate was low and PFS as well as OS were shorter in patients treated with a study drug than in patients treated based on physician’s choice while in study.

In the first half of the trial when tumor sampling was based on biopsy, only 32% of patients had results available to the MTB within 28 days from inclusion. This proportion improved considerably to 75% in the second half of the trial with sampling by ctDNA. Expediting this step, e.g. by tumor sampling and analysis in parallel with start of last line therapy, is central to minimizing the risk of disease progression beyond the treatment window while waiting for genomics analysis results. Since ctDNA analysis has been validated for analysis of tumor genomics [22] it is suggested to be a promising approach for genomics-guided PCM [23] although our observation that 22% of the analyses were considered inconclusive is a caveat.

Regarding the modest activity of the genomics-guided treatment selection, one explanation is that most patients recruited were heavily pretreated and, thus, at increased risk of being multidrug resistant. Another probable explanation is the revised and broader criteria for study drug allocation in the second half of the study. Although the expanded criteria were based on the current understanding of the cancer genome and drug pharmacodynamics [20], moving further down in the hierarchy of actionable targets according to the ESCAT tiers [2] will increase the risk of lack of drug effect [24, 25]. From these perspectives, the genomics-guided non-comparative PCM trials recruiting last line patients ongoing or about to start [26, 27] might need consideration.

An evident limitation in MEGALiT was the low number of targeted drug available within the trial. A considerably greater armamentarium of targeted drugs had opened for more patients getting access to a matched treatment and could also provide benefit from mechanistically based drug combinations as illustrated by the efficacy of cobimetinib combined with vemurafenib in *BRAF* mutated colorectal cancer [28].

As the study recruited patients after their last line of standard therapy, it was unexpected that almost half of the patients who were not allocated a study drug were treated. These patients had significantly longer PFS and OS while receiving treatment of physician’s choice in study, and a greater fraction had a PFS ratio of ≥ 1.3 compared with patients treated with a study drug, suggesting that the physician’s choice of treatment was better than genomics-guided treatment selection. However, although the two groups of patients were similar at baseline they differed with respect to cancer diagnosis and patients treated with physician’s choice may have had prognostic features associated with a better outcome at start of treatment. Furthermore, in these patients the interval between radiological assessments of tumor response may have been longer than for patients on a study drug, resulting in PFS inflation. Another study limitation for this group of patients was that data on treatment and outcome were collected from patient files without renewed formal monitoring.

In addition, the genomics analysis results seemingly influenced the physician’s choice of treatment in patients not allocated study drug treatment. Thus, in practice, the MEGALiT trial could be regarded as a genomics-guided treatment trial with access, yet uncontrolled, to a broad panel of targeted drugs beyond the last line of standard therapy. In this perspective, it could be observed that PFS and OS in MEGALiT for patients treated while in trial were similar or slightly inferior to the outcomes in comparable trials [3, 7, 9, 10, 16, 17]. The superior disease control rate, PFS and OS in the Drug Rediscovery Protocol (DRUP), used as a template for the MEGALiT trial, might be explained by its inclusion of a cohort of patients with mismatch repair deficient tumors treated with anti PD-1 monotherapy, fewer lines of treatment prior to inclusion and access to a greater number of drugs [3].

In contrast, patients who were not treated within the trial had poor prognostic features with respect to performance status, fewer lines of treatment prior to inclusion, shorter PFS on the treatment immediately prior to study inclusion, as well as more difficult-to-treat cancer diagnoses with poor prognosis. This underscores the impact of patient selection on the trial outcome, limiting between group comparisons.

Overall, the experience so far from genomics-guided single drug therapy beyond ESCAT tier 1 suggests that this approach does not provide obvious patient benefits in heavily pretreated patients, a conclusion also supported by the few randomized controlled trials published [14–16]. A way forward might be to target as many gene mutations as possible using drug combinations based on the genomic analysis findings [9]. However, the treatment of patients with drug combinations not yet investigated in phase 1 trials poses problems from pharmacokinetic, safety, and regulatory perspectives [29]. Other suggestions to improve future clinical trials of the genomics-guided PCM approach are to have broad access to targeted drugs and to move the trials to patients in earlier lines of treatment [30].

From a general perspective, the PCM concept would need to consider not only genomics but also other biomarkers of potential importance for treatment outcome. This includes proteomics, transcriptomics, epigenomics, metabolomics, tumor immune scores, patient microbiota, phenotypical drug sensitivity testing, and therapeutic drug monitoring to optimize drug dosing [31–33]. Such a multi-factorial concept will pose a formidable challenge when it comes to data collection, curation, analysis, and interpretation, and will require advanced, artificial intelligence-driven bioinformatical analysis.

In conclusion, we found genomics-guided PCM feasible and safe in patients with advanced treatment-refractory solid tumors, although it was difficult to evaluate patient benefits. Access to a greater number of targeted drugs, use of drug combinations, moving the concept to earlier lines of treatment, and incorporation of information from additional types of biomarkers could enhance the benefit of PCM.

## Supplementary Material

Feasibility and outcome of genomics-guided treatment selection in advanced cancer – the MEGALiT explorative clinical trial

## Data Availability

Data are available from the authors upon reasonable request.
