# Supplementary material for: Feasibility and outcome of genomics-guided treatment selection in advanced cancer – the MEGALiT explorative clinical trial
Source: Acta Oncol. 2025 Jun 4;64:43366. doi: 10.2340/1651-226X.2025.43366 (PMC12160592; doi:10.2340/1651-226X.2025.43366)
Supplement: Feasibility and outcome of genomics-guided treatment selection in advanced cancer – the MEGALiT explorative clinical trial [file AO-64-43366-s1.pdf]

Supplementary material has been published as submitted. It has not been copyedited, or typeset by Acta Oncologica

Supplementary Table 1. Consensus pathway component changes after protocol update for allocation of treatment with everolimus, cobimetinib, and niraparib*.

	Everolimus	Cobimetinib	Niraparib
Mutations in the genes indicated for allocation of study drug	*PTEN, INPP4B, PIK3R1-3, PIK3CA/B, AKT1-3, PPP2R1A, TSC1/2, STK11, RHEB, RICTOR, RPTOR, MTOR*	*ERBB1-4, MET, PDGFRA, FGFR1-4, KIT, IGF1R, RET, ROS1, ALK, FLT3, NTRK1-3, JAK2, CBL, ERRFI1, ABL1, SOS1, NF1, RASA1, PTPN11, KRAS, HRAS, NRAS, RIT1, ARAF, BRAF, RAF1, MAP2K1-2*	*BRCA1, BRCA2, ATM, BRIP1, BARD1, CDK12, CHEK1, CHEK2, FANCL, PALB2, PPP2R2A, RAD51B, RAD51C, RAD51D, RAD54L*, high HRD score

*General criteria for the cobimetinib and everolimus were revised with a new definition; (i) pathogenic or likely pathogenic genetic aberration in a consensus pathway component that is predicted to activate the signaling or (ii) a pathogenic or likely pathogenic genetic aberration in a gene that is not a consensus pathway component of signaling pathway, but where a firm body of evidence suggests that it leads to activation. For niraparib treatment criteria were aberrations linked to “homologous repair deficiency” (HRD) that are part of current FDA approved molecular indications for PPARi in specific cancer types, i.e. ovarian carcinoma and prostate carcinoma. Furthermore, for everolimus, cobimetinib and niraparib the following general criteria for treatment allocation should apply; (i) no identified literature suggesting that the drug lacks effect or impacts negatively on patient outcome in the treatment of patients with tumors of the entity in question, (ii) literature supporting benefit of the drug in treatment of patients with the same or similar tumor type or, for atezolizumab (iii) literature supporting benefit of monotherapy in at least a subset of genetically uncharacterized patients, with the same or similar tumor type.

Supplementary Table 2. Physician's choice of treatment in patients not allocated to a study drug but treated while in study.

**Cancer drug based on mechanism of action***	**Patients; n**
Cytotoxic drug single	7
Cytotoxic drug combination	5
Anti-VEGF, single/combination	4
mTOR-inhibitor, single/combination	4
MEK-inhibitor, single/combination	4
PARP-inhibitor, single/combination	3
CDK-inhibitor	3
Anti-estrogen/anti-androgen	7
Immune check-point inhibitor	8
Multi-kinase inhibitor	1
Antibody drug conjugate	1
Other	8

Supplementary Table 3. Molecular biomarkers used for allocation of study drug in the individual patients (n=38) starting treatment.

Study drug	Diagnosis	Molecular biomarker
Atezolizumab (n=18)	CRC*	TMB 8; 18; 8; 13
	Esophagus	TMB 11
	Gastric	TMB 25
	NEC	TMB 10
	NET	TMB 34
	Ovarian	TMB 9; 10; 13; 15
	Prostate	TMB 8
	Uterine body	TMB 11; 9
	Uterine cervix	TMB 9; 13; 9,
Cobimetinib (n=13)	Breast	*RAF1 mut*
	CRC	*GNAS R201H; NF1;*
	Melanoma	*NF1; GNAQ*
	Ovarian	*BRAF mut; KRAS mut 12; FBXW7/KRAS G12V; SOS1 mut*
	Parotid	*KRAS mut/PIK3CA mut*
	Prostate	*NF1; MET ampl*
	Vaginal	*NF1*
Niraparib (n=5)	Breast	*ATM loss*
	Cholangiocellular	*ATM mut*
	Glioma	*ATM mut*
	NET	*BRCA1/CHEK2*
	Mesothelioma	*BAP1*
Everolimus (n=2)	NET	*TSC2*
	Uterine cervix	*PIK3CA mut*

Abbreviations: CRC, colorectal cancer; TMB, tumor mutational burden (number/megabase); NET, neuroendocrine tumor; NEC, neuroendocrine cancer; ampl, amplification; mut, mutation.

Supplementary Table 4. Best overall response on study drug treatment.

Basket/Drug	Best overall response rate on treatment allocated; n, (%) and DC16w*
All drugs	CR: 0<br>PR: 2 (6)<br>SD: 7 (20)<br>PD: 23 (66)<br>DC16w: 8 (23)<br>NE: 3 (9)
Atezolizumab	PR: 1 (6)<br>SD: 1 (6)<br>PD: 14 (82)<br>DC16w: 2<br>NE: 1 (6)
Cobimetinib	PR: 1 (8)<br>SD: 4 (33)<br>PD: 5 (42)<br>DC16w: 4<br>NE: 2 (17)
Niraparib	PR: 0<br>SD: 2 (40)<br>PD: 3 (60)<br>DC16w: 2<br>NE: 0
Everolimus	PR: 0<br>SD: 0<br>PD: 1 (100)<br>DC16w: 0<br>NE: 0

*Abbreviations; DC16w, disease control ≥ 16 weeks (including PR and SD); CR, complete response; PD, progressive disease; PR, partial response; SD, stable disease; NE, not evaluable due to early clinical deterioration after the start of treatment.
